# Women Veterans’ experiences discussing household firearms with their intimate partners: collaborative, devalued, and deferential relational types

**DOI:** 10.1186/s40621-023-00452-7

**Published:** 2023-07-31

**Authors:** Evan R. Polzer, Carly M. Rohs, Suzanne M. Thomas, Ryan Holliday, Christin N. Miller, Joseph A. Simonetti, Katherine M. Iverson, Lisa A. Brenner, Lindsey L. Monteith

**Affiliations:** 1 VA Rocky Mountain Mental Illness Research, Education and Clinical Center for Suicide Prevention, Aurora, CO USA; 2 Department of Psychiatry, University of Colorado Anschutz Medical Campus, Aurora, CO USA; 3 Division of Hospital Medicine, University of Colorado Anschutz Medical Campus, Aurora, CO USA; 4 Women’s Health Sciences Division, National Center for PTSD, VA Boston Healthcare System, Boston, MA USA; 5 Department of Psychiatry, Boston University Chobanian and Avedisian School of Medicine, Boston, MA USA; 6 Department of Physical Medicine and Rehabilitation, University of Colorado Anschutz Medical Campus, Aurora, CO USA; 7 Department of Neurology, University of Colorado Anschutz Medical Campus, Aurora, CO USA

**Keywords:** Veteran, Firearm, Suicide, Women, Lethal means safety counseling, Intimate partner

## Abstract

**Background:**

Rates of firearm suicide have increased among women Veterans. Discussing firearm access and reducing access to lethal means of suicide when suicide risk is heightened are central tenets of suicide prevention, as is tailoring suicide prevention strategies to specific populations. While research has begun to explore how to optimize firearm lethal means safety counseling with women Veterans, there is limited knowledge of women Veterans' perspectives on including their intimate partners in such efforts. This gap is notable since many women Veterans have access to firearms owned by other household members. Understanding women Veterans’ experiences and perspectives regarding including their partners in firearm lethal means safety conversations can provide important information for tailoring firearm lethal means safety counseling for women Veterans.

**Methods:**

Qualitative interviews were conducted with 40 women Veterans with current or prior household firearm access. Interview questions focused on the roles of women Veterans’ partners in household firearm access and storage, as well as women Veterans’ perspectives regarding including intimate partners in firearm lethal means safety counseling. Inductive thematic analysis was performed.

**Results:**

Three relational types characterized how household firearms were discussed between women Veterans and their partners: *collaborative, devalued,* and *deferential*. These types were distinguished via women Veterans’ agency in decision-making related to household firearms, partners’ receptivity to women Veterans’ mental health or trauma histories, and willingness (or lack thereof) of partners to change household firearm access and storage considering such histories. Intimate partner violence was common in the devalued relational subtype.

**Conclusions:**

Findings extend knowledge regarding the context of women Veterans’ household firearm access, including relational dynamics between women Veterans and their partners. The acceptability, feasibility, challenges, and facilitators of including women Veterans’ partners in firearm lethal means safety efforts likely vary for each relational type. For example, in dyads with a collaborative dynamic, incorporating partners may create opportunities for increased firearm safety, whereas including partners in devalued dynamics may present unique challenges. Research is warranted to determine optimal methods of navigating firearm lethal means safety counseling in the presence of each relational dynamic.

## Background

The Department of Veterans Affairs (VA) has prioritized suicide prevention as its leading clinical priority, and there has been increased recognition and concern regarding suicide among women Veterans (Denneson et al. 2021; Hoffmire et al. 2021; Monteith et al. 2022a). In 2020, women Veterans experienced an age-adjusted suicide rate double that of women non-Veterans, with rates of 14.7 and 6.8 per 100,000, respectively (U.S. Department of Veterans Affairs and Office of Mental Health and Suicide Prevention 2022a). Suicide rates among women Veterans were further elevated among those using Veterans Health Administration (VHA) services, with an age-adjusted suicide rate of 16.8 per 100,000 in 2020 (U.S. Department of Veterans Affairs and Office of Mental Health and Suicide Prevention 2022b).

An important component to preventing suicide entails understanding the methods used in suicide deaths within a specific population. Among women Veterans who die by suicide, firearms have become the most common suicide method, used in nearly half (48.2%) of suicide deaths in 2020 (U.S. Department of Veterans Affairs and Office of Mental Health and Suicide Prevention 2022a). Notably, use of firearms as a suicide method has increased among women Veterans, despite *decreasing* among non-Veteran women and increasing to a lesser extent among Veteran men (U.S. Department of Veterans Affairs and Office of Mental Health and Suicide Prevention 2022a). These findings suggest the import of preventing firearm-related suicide among women Veterans.

Decreasing access to firearms and other potentially lethal means of suicide during periods of heightened suicide risk is recommended by numerous institutions and agencies (Office of the Surgeon General 2012; U.S. Department of Veterans Affairs and Office of Mental Health and Suicide Prevention 2018). Additionally, recent White House suicide prevention memoranda have emphasized lethal means safety, including training healthcare providers to implement lethal means safety counseling (LMSC) and increasing awareness among Veterans and their families (White House 2021). When working with Veterans at increased suicide risk, VHA providers are expected to assess patients’ access to firearms and work collaboratively with them to engage in LMSC—key components of suicide risk assessment and prevention. Importantly, suicide prevention strategies recommended by the White House include “tailoring solutions to sub-populations where possible,” highlighting the need to respond to “the unique needs and contexts” of different populations (p. 10) (White House 2021). Despite this, there is limited knowledge to inform firearm LMSC for women Veterans. Consequently, there have been increasing calls to bolster knowledge that would inform a tailored approach to firearm LMSC with women Veterans (Monteith et al. 2022a; Spark et al. 2022).

One important gender difference integral to informing firearm LMSC strategies entails the sources of women Veterans’ firearm access. While Veteran men’s firearm access is nearly exclusively through personal firearm ownership, women Veterans’ firearm access tends to also occur through other household members. Cleveland and colleagues found that Veteran women were less likely than Veteran men to personally own firearms (24.4% vs. 47.2%, respectively), but more likely to report living in households with firearms that they did not personally own (14.4% vs. 2.2%) (Cleveland et al. 2017). Subsequent surveys with post-9/11 women Veterans also reported a high prevalence of personal and household firearm ownership among women Veterans (Monteith et al. 2022b, Monteith et al. 2023). Further, a qualitative study highlighted the prominent role of spouses and partners of women Veterans in household firearm access (Monteith et al. 2020)—findings which converge with survey-based findings in which married women Veterans reported a higher prevalence of living in a household with firearms owned by someone else (Monteith et al. 2022b). As such, firearm LMSC approaches that assume women Veterans are the owners and primary decision makers regarding household firearms may be insufficient for reducing their access to firearms when they are at elevated risk for suicide, as other household members may be responsible for such decisions.

Unfortunately, there is limited knowledge regarding how household firearm access (e.g., acquisition, storage) is decided and discussed between women Veterans and their partners. Further, intimate partner violence (IPV; i.e., physical, psychological, and sexual aggression) is experienced by 60% of partnered women Veterans (Iverson et al. 2017) and may complicate firearm LMSC. Understanding ways to facilitate firearm LMSC when household firearms are owned by a partner, including when IPV is present, is essential to prevent firearm injuries and deaths among women Veterans. However, to our knowledge, no studies have investigated women Veterans’ perspectives regarding involving their partners in firearm LMSC, nor their perspectives on addressing firearm LMSC when IPV, or other relational factors that may complicate LMSC, are present.

Given these knowledge gaps, the current study sought to understand women Veterans’ experiences and perspectives regarding the roles of their partners in household firearm access and storage, and the relational dynamics in which women Veterans and their partners make decisions about and discuss household firearms. We also explored women Veterans’ perspectives regarding involving their partners in firearm LMSC.

## Methods

### Sampling

Methods for this study, which examined women Veterans’ perspectives, experiences, and preferences for discussing firearms with healthcare providers, have been reported previously (Polzer et al. under review). We enrolled women Veteran participants in this study between April 2021 and January 2022. Individuals were eligible to participate if they met all of the following criteria: reported self-identifying as a woman or that their birth sex was female; previously served on active duty in the U.S. military; were 18–89 years of age; lived in a household with firearm(s) present and/or personally owned firearm(s) at any point after separating from military service; were eligible to receive VHA services and had used VHA services (ever); had experienced lifetime suicidal ideation and/or suicide attempt(s), as assessed by an abbreviated version of the Self-Injurious Thoughts and Behaviors Interview (SITBI; Nock et al. 2007); and were able to provide informed consent. Those currently active duty or mobilized on Reserve or National Guard duty, or determined to currently be at high acute risk for suicide by a licensed psychologist on the study team, were not eligible to participate.

To recruit our sample, we used the VA Corporate Data Warehouse (CDW) to identify a national sample of women Veterans who had enrolled in and used VHA outpatient care. We mailed each an invitation letter inviting them to contact us if interested in participating. We supplemented this recruitment strategy with advertising in VHA facilities and at community events, snowball sampling, and mailing an invitation letter to women Veterans who participated in prior research at our center and agreed to be contacted about subsequent research opportunities. We took a purposeful sampling approach to obtain a sample that was demographically and geographically diverse. Throughout data collection, our team monitored sample characteristics, noting any gaps in sample composition and attempting to address these in subsequent recruitment efforts. Thus, sampling emphasized thematic data saturation (when no novel data are yielded from additional interviews) and purposeful sampling. Enrollment concluded after we reached saturation and were satisfied that we had addressed purposeful sampling goals.

### Procedures

Interested individuals were screened for eligibility by phone (*n* = 79). Of those, 43 (54.4%) were eligible and 36 (45.6%) were ineligible. Reasons for ineligibility included not having a history of lifetime suicidal ideation or suicide attempt (*n* = 22; 61.1%), lacking a history of personal or household firearm access (*n* = 11; 30.6%), never having used VHA care (*n* = 2; 5.6%), or currently being mobilized on Reserve duty (*n* = 1; 2.8%). Of individuals eligible at screening, 40 (93.0%) participated.

### Study appointments

Appointments were conducted virtually and/or by telephone and began with obtaining informed consent, followed by the University of Washington Risk Assessment Protocol (UWRAP; Linehan et al. 2000) pre-assessment to assess current levels of stress, suicidal urges, and intent to harm oneself, to confirm appropriateness to participate. This was followed by trained research staff (LLM, RH, or SMT) conducting the audio-recorded qualitative interview, using the semi-structured interview guide developed by our team for this study (Polzer et al. under review). Thereafter, staff administered an abbreviated version of the SITBI (Nock et al. 2007) to assess suicidal ideation and attempt history. Staff also administered self-report measures, and those relevant to the current study are described below. To screen for lifetime and past-year IPV, we administered the Extended-Hurt, Insult, Threaten, Scream (E-HITS; Chan et al. 2010), which has demonstrated acceptable reliability and validity with women Veterans (Iverson et al. 2015). A positive screen was operationalized as a score > 7 (Iverson et al. 2015). We also assessed military sexual trauma history using the standard VA Military Sexual Trauma Screening Questions (U.S. Department of Veterans Affairs and Veterans Health Administration 2018), which has demonstrated construct validity (Mengeling et al. 2019). Further, we administered brief screening measures of possible posttraumatic stress disorder (PTSD) (Primary Care PTSD Screen for DSM-5; Prins et al. 2016), depression (PHQ-2; Kroenke et al. 2003), and substance misuse (CAGE-AID; Brown et al. 1998). These measures have also demonstrated adequate psychometric performance, including internal reliability and validity (Bovin et al. 2021; Staples et al. 2019; Brown and Rounds 1995). Additionally, we assessed demographic characteristics (Demographics Module; Behavioral Risk Factor Surveillance System 2018 Demographics; Centers for Disease Control and Prevention 2018 and National Health and Nutrition Examination Survey, 2013–2014; Centers for Disease Control and Prevention, National Center for Health Statistics 2013) and military service history (PhenX Toolkit: Military Service Demographics Protocol; Hamilton et al. 2011). Staff concluded the appointment with the UWRAP post-assessment (Linehan et al. 2000) and debriefing, reviewing relevant safety-related information and resources with the participant. Participants received $50 for their participation in this IRB-approved study.

### Analytic process

An inductive approach to coding and analysis was used to obtain a rich understanding of women Veterans’ experiences and perspectives. A constant comparative method, a form of latent content analysis, was used to analyze transcripts (Hewitt-Taylor 2001). This approach facilitated identification of meaningful themes (Hsieh and Shannon 2005). Prior to data collection and analysis, all coders engaged in bracketing (i.e., acknowledging their pre-existing biases and knowledge regarding the topic and discussing ways in which biases could potentially impact interpretation) (Carpenter et al. 1999). Reflexivity was discussed (Berger 2015). ATLAS.ti v22.2.4 was used for coding and analysis. Quantitative analyses (i.e., descriptive characteristics of the sample) were performed using SAS, v9.4.

Initial transcripts were independently reviewed by two team members (ERP, CMR), who inductively identified a set of codes based on immersing themselves in transcripts and interview notes. Next, the team collaboratively developed an initial codebook that included codes and their definitions, then independently applied the codes to a subsequent set of transcripts and notes. The team met again to collaboratively reconcile coded transcripts and adapt the codebook as needed. Analysis was iterative, re-visiting the data to connect it with emerging insights (Srivastava and Hopwood 2009). Consensus meetings occurred to deliberate disagreements or conflicts in code meaning or application, resolving any disputes among coders. Sixty percent of transcripts were double-coded, with the remaining 40% coded by a single coder, resulting in 100% of transcripts coded. COREQ standards for reporting qualitative findings were followed (Tong et al. 2007).

## Results

### Sample descriptives

#### Sociodemographics and military service history

All 40 participants identified as cisgender women. Participants ranged in age from 24 to 73 (median = 53). As reflected in Table 1, the majority of participants were currently (i.e., at the time of the interview) either married or a member of an unmarried couple (42.5%) or separated or divorced (42.5%). Fewer than half (42.5%) currently lived with a partner. The majority identified their sexual orientation as heterosexual (72.5%), and smaller percentages identified as lesbian or gay (12.5%) or bisexual (12.5%). Only a minority currently lived with (17.5%), or had current parenting responsibilities for (15.0%), anyone under age 18. Rurality and urbanicity varied considerably. The largest proportions of participants resided in the South or West (42.5% each), although participants from the Midwest (10.0%) and Northeast (5.0%) were also included. Approximately two-third of participants self-identified their race as White (65.0%), nearly one-fourth (22.5%) as Black, and smaller proportions as American Indian or Alaska Native (12.5%) or Asian (2.5%). One-tenth identified their ethnicity as Hispanic (10.0%). Education varied broadly. Regarding current employment, the largest proportions of participants reported being disabled (42.5%) or working (37.5%).

**Table 1:** Sociodemographics and military service histories (*N* = 40)

	*n*	%
*Marital status*		
Married/couple	17	42.5
Separated/divorced	17	42.5
Never married	4	10.0
Widowed	2	5.0
*Lives with partner*		
Yes	17	42.5
No	23	57.5
*Sexual orientation*		
Heterosexual	29	72.5
Lesbian/gay	5	12.5
Bisexual	5	12.5
None	1	2.5
*Lives with minor*		
Yes	7	17.5
No	33	82.5
*Parenting responsibilities*		
Yes	6	15.0
No	34	85.0
*Rurality*		
Rural	8	20.0
Small town	11	27.5
Medium-sized town	4	10.0
Suburb	3	7.5
City	14	35.0
*Region*		
South	17	42.5
West	17	42.5
Midwest	4	10.0
Northeast	2	5.0
*Race*^a^		
White	26	65.0
Black	9	22.5
American Indian/Alaska Native	5	12.5
Asian	1	2.5
*Ethnicity*		
Non-Hispanic	36	90.0
Hispanic	4	10.0
*Education*		
High school or less	3	7.5
Some college	9	22.5
Associate’s degree	7	17.5
Bachelor’s degree	9	22.5
Master’s degree or higher	12	30.0
*Employment*		
Disabled	17	42.5
Working	15	37.5
Retired	5	12.5
Other^b^	6	15.0
*Service era*^a^		
Post-Vietnam/Peacetime	20	50.0
Desert Storm/Desert Shield	19	47.5
OEF/OIF/OND	16	40.0
Vietnam	3	7.5
*Military branch(es)*^a^		
Army	21	52.5
Air Force	9	22.5
Navy	6	15.0
Marine Corps	6	15.0
*Military rank*		
Enlisted	33	82.5
Officer	7	17.5

Responses not endorsed by any participants (e.g., Native Hawaiian/Pacific Islander race, military service before 1964) are not displayed

*OEF/OIF/OND* Operation Enduring Freedom/Operation Iraqi Freedom/Operation New Dawn

^a^Totals could exceed 100% as response options were not mutually exclusive

^b^Included temporarily laid off, seeking employment, on maternity leave, and students

Participants reported military service during Post-Vietnam/Peacetime (50.0%), Desert Storm or Desert Shield (47.5%), Operations Enduring Freedom, Iraqi Freedom, or New Dawn (40.0%), and the Vietnam War (7.5%). Army was the most common branch of service (52.5%), followed by Air Force (22.5%), Navy (15.0%), and Marine Corps (15.0%). The majority (82.5%) held enlisted rank at separation.

#### Firearms

All participants reported currently (60.0%) or previously (40.0%) having household firearms (Table 2). The sample varied regarding personal firearm ownership, with the largest percentage (42.5%) currently owning firearms, 37.5% never owning firearms, and 20.0% previously owning firearms. The sample was similarly comprised of participants who reported that other household members currently (42.5%) or never (57.5%) owned firearms.

**Table 2:** Household firearms (*N* = 40)

Characteristic	*n*	%
*Household firearm(s)*^a^		
Current^b^	24	60.0
Past	16	40.0
*Personal firearm ownership*		
Yes, currently	17	42.5
Yes, previously	8	20.0
No, never	15	37.5
*Anyone else in household currently owns firearm(s)*		
Yes	17	42.5
No	23	57.5

^a^Reflected if there were currently firearms in the household, owned by the participant and/or another household member

^b^Participants who reported both current and past household firearms were categorized as “current”

#### Suicidal thoughts and behaviors, interpersonal violence, mental health, and healthcare

All participants (100%) reported experiencing lifetime suicidal ideation, and half (50.0%) reported experiencing lifetime suicide attempt(s) (Table 3). Nearly two-thirds (62.5%) had considered firearms as a suicide method. Screeners administered during the study indicated that most participants had experienced military sexual trauma (85.0%) and lifetime IPV (84.6%). Ten percent (10.3%) had experienced IPV in the past year. Additionally, the majority screened positive for current posttraumatic stress disorder (85.0%) and depression (66.7%). Over one-third (38.5%) screened positive for substance misuse. Most (85.0%) had used VHA care in the past year. Further, nearly all (95.0%) had used mental health services in their lifetime, including in the past year (71.1%).

**Table 3:** Suicidal thoughts and behaviors, interpersonal violence, mental health, and healthcare USE (*N* = 40)

Characteristic	*n*	%
*Suicidal ideation and attempt*		
Lifetime suicidal ideation	40	100.0
Lifetime suicide attempt(s)	20	50.0
Firearms considered during suicidal ideation	25	62.5
*Interpersonal violence*		
Military sexual trauma^a^	34	85.0
IPV^b,c^	33	84.6
*Mental health screens*^d^		
Posttraumatic stress disorder	34	85.0
Depression^c^	26	66.7
Alcohol/drug misuse^c^	15	38.5
*Healthcare*		
Use of VHA services in past year	34	85.0
Lifetime mental health care^e^	38	95.0
Past-year mental health care^f^	27	71.1

*IPV* intimate partner violence, *VHA* Veterans Health Administration

^a^Military sexual harassment (*n* = 34; 85.0%); military sexual assault (*n* = 24; 60.0%)

^b^Results reflect IPV screening results; 4 of the 39 participants with IPV data available (10.3%) screened positive for IPV in the past year

^c^*n* = 39

^d^Reflects screening results suggestive of possible diagnoses

^e^Included outpatient (*n* = 36; 94.7%) or inpatient (*n* = 14; 36.8%) services

^f^*n* = 38

### Navigating firearm access and storage with partners: relational types

Decisions and discussions between the Veteran and her partner regarding household firearm access and storage could be characterized in three ways, as *collaborative*, *devalued*, or *deferential.* Here, we briefly summarize each of these, then describe in greater depth the factors that constitute each relational dynamic, using quotes from women Veterans to describe each.

#### Collaborative

For many women Veterans, the role of their partners in household firearm decisions could be characterized as *collaborative*. In these cases, women Veterans described having both a voice and a sense of agency in firearm matters, feeling highly involved in decisions about household firearms, whether their own or their partner’s. In these cases, Veterans felt that they could engage in equal, shared decision-making with their partner regarding firearms in the home. They felt that both parties were equal in their ability to express their preferences, needs, and concerns regarding firearm access and storage. Generally, Veterans and their partners in this group both personally owned and had access to household firearms. Further, these collaborative relationships were exemplified by the partner’s willingness to make changes to household firearm access, storage, or use. Such changes seemed to be driven by three types of concerns from the woman Veteran: (1) general apprehensions about household firearms; (2) concern that her mental health or trauma history rendered firearm access a danger to herself; and (3) concerns about the safety of children or grandchildren in the home. These collaborative interactions were characterized by high levels of trust between the woman Veteran and her partner, and these Veterans described their partners as current or potential collaborators in firearm LMSC.

#### Devalued

In stark contrast, many women Veterans described their role in shaping decisions regarding household firearms with their partner as *devalued*. These interactions were characterized by the Veteran having little to no role in the decision-making process surrounding household firearms, despite having prominent concerns regarding their presence, use, or storage in the home. In such instances, the partner used or stored household firearms autonomously without seeking or considering input from the Veteran. Sometimes, women Veterans in these circumstances voiced repeated objections or concerns, yet there typically were not active discussions about firearms, and partners were unresponsive to the Veteran’s concerns and unwilling to change how firearms were used and stored (e.g., reduce accessibility). The partners of women Veterans in the devalued group most often were the owners of household firearms; however, in a few instances, women Veterans themselves also owned firearms (in some instances, they were forced to do so by their partner, due to their partner being unable to lawfully own or purchase one). Partners dismissed, downplayed, or directly contributed to the mental health concerns or trauma histories of the woman Veteran that made firearm access dangerous. Such relationships were often characterized by IPV, with the partner maintaining firearms, in part, as a means of control and intimidation. The presence of children had little impact on changing the status of household firearms. Devalued relational dynamics were characterized by low levels of trust between the woman Veteran and her partner. The involvement of such partners in firearm LMSC was low and perceived by woman Veterans as undesired, inherently problematic, and likely unsafe due to IPV and the partner’s domineering behavior.

#### Deferential

Some women Veterans’ attitudes and roles toward household firearms were noted as *deferential.* These relationships were characterized by a high degree of trust, which was paramount. Specifically, the woman Veteran afforded her partner almost unilateral control over household firearms, deferring nearly all decisions about where, why, and how firearms were used and stored in the home. This high level of trust manifested through the Veteran and her partner having nearly unstated, implicit mutual understanding about firearm use and storage. Most typically, the woman Veteran’s partner was the owner of household firearm(s). Despite deferring household firearm decisions to their partners, these women Veterans still felt empowered to voice concerns about their partners’ firearms and to be agentic in doing so if they perceived that they needed to discuss potential changes. For example, these women Veterans expressed that although they were largely satisfied with current household firearm storage practices and use, they were confident that their partner would make changes to household firearms in the future if the woman Veteran voiced concerns due to her mental health, trauma history, or the presence of children (especially young children) in the home. They indicated that their partners had been (or would be) receptive and open to making changes to how and where they stored or used their firearms. They expressed openness to include such partners in LMSC efforts, due to the high trust that characterized such relationships.

### Characteristics of firearm dynamics between women Veterans and their partners

In what follows, we describe each factor that differentiated relational types in greater detail. These factors provide important insights regarding the context and dynamics of these relationships and how partners may, or may not, be appropriate collaborators in firearm LMSC. Synopses of these factors can be found in Table 4.

**Table 4:** Characteristics that distinguish each firearm decision-making relational type between woman Veterans and their partners

Relational type	Factors/dynamics					
	Woman Veteran’s desired degree of involvement	Woman Veteran’s voice or agency in firearm decisions	Partner receptivity to the mental health and trauma history of the women Veteran	Impact of presence of children or grandchildren	Partner willingness to change firearm storage or use	Woman Veteran’s desire to include partner in firearm LMSC
Collaborative	Active, engaged, motivated	Agentic, optimistic, confident	Responsive, attuned, respectful	Positive, motivates change	High	High, if not already being done
Devalued	Overruled, disregarded, minimized	Denied, ignored, gaslit	Indifferent, contributing, disrespectful	Dismissive, underplays seriousness	Low, unwilling, contemptuous	Low, especially in context of IPV
Deferential	Passive, civil, indifferent, due to high level of trust in partner	Assured, hopeful, confident	Understanding, open, compassionate	Hypothetical, would motivate action	Hypothesized as high, trust in partner to do so	High, albeit hypothetical

*IPV* intimate partner violence, *LMSC* lethal means safety counselling

#### Women Veterans’ desired degree of involvement

The first matter to consider is the degree to which a woman Veteran desired to be involved in decision-making regarding household firearms. As reflected by the different relational types, women Veterans were not uniform in their stated desire to be involved in decisions regarding firearms.

##### Collaborative

Women Veterans in the *collaborative* group desired active involvement in household firearm decisions and felt highly engaged in discussing household firearms with their partners. For example, a Veteran stated that she and her wife were “pretty much on the same page on where to keep [the firearm] and the security of it,” adding that if she wanted to make changes to how it was stored, her wife “would be open to that as well.” Whether because household firearms tended to be jointly owned, or due to the nature of their relationship with their partner, women Veterans in collaborative relationships desired to be actively involved in decisions regarding how firearms were stored within their homes, both currently and in the future.

##### Devalued

Women Veterans in *devalued* relationships also had a desire to be highly involved in firearm discussions, expressing a keen and sharp drive to be more involved in decisions about how household firearms were used and stored. However, they were largely not afforded this opportunity. In instances in which household firearms were discussed, women Veterans noted that they were often overruled or disregarded. Summing up this tension, one woman explained how it is “so challenging because there’s such a huge dynamic in a relationship when someone has a deadly weapon in the home. And that’s even more so when it's against your wishes. You would prefer for that weapon to not be there, but yet they are, it's still there. And you definitely don’t feel comfortable to speak up.”

##### Deferential

In direct contrast to the high actual or desired involvement of women Veterans in the aforementioned groups, women Veterans in the deferential group were largely *deferential* to their partner on decisions regarding household firearms. The main distinction between Veterans in the deferential and devalued groups was that women Veterans in the deferential group appeared to be satisfied with this arrangement, as their high degree of trust in their partners led them to defer such decisions to their partners. One Veteran noted that although she trusted her husband and “likes the fact that [his firearms are] in the household where it’s easy to access in case something were to happen,” she generally tries not to get too involved. “I know how to use it, I just don’t wanna touch it,” she explained, later noting how her husband “asks me if I wanna practice or use it real quick to see how everything works. I’m like ‘I know how it works, I’m fine.’”

#### Women Veterans’ voice or agency in firearm decisions

Related to their desired level of involvement with household firearms was the extent to which women Veterans had their voices heard (or expected that their perspective would be heard and considered) and had an agentic role in household firearm decisions. If a Veteran wanted to be involved in these decisions, how much was her voice valued? Did she have agency in shaping household firearm use, storage, or accessibility with her partner? These questions highlight further nuances within dyads.

##### Collaborative

*Collaborative* relationships were predicated on the Veteran having a high degree of agency in such discussions, feeling free to voice her concerns, with partners being receptive and open to discussing and problem-solving such concerns. One woman described this process of mutual understanding and problem-solving. “I just told her because of my mental illness, I can’t trust myself, let alone trust myself with guns. Not that I’m suicidal all the time, I mean I have been suicidal in the past. But at that point, it becomes an impulsive act.” She continued by explaining how “we talked about it and I told her that, and she agreed, and she understands, and she’s doing what she can to keep things safe.”

##### Devalued

Conversely, Veterans in *devalued* relationships described the opposite: having their voices denied, gaslit, or otherwise ignored. “He just brought [a firearm] home and said ‘I bought a gun today.’ And I said ‘Cool, please don’t shoot me.’ And he put it in his nightstand and that was it,” one woman explained, describing her ex-husband’s ignoring of her objections about having firearms in the home. Further, another Veteran described how, despite having concerns about having firearms in the home, her voice was minimized and disregarded, and her husband “would just do his thing, ‘cause we were living in his house.” One Veteran stated that she had limited agency in the firearm decision-making process, detailing how it was “more of him telling me what to do and me not being in a positive state of mind and just going with it. Just whatever he said, whatever he was doing, I was just kind of like ‘Okay’ and not standing up for myself.” Of note, many Veterans described these instances of having their voiced denied in tandem with instances of IPV. In such instances, firearms were often used against women Veterans as instruments of intimidation, control, and manipulation. For example, when asked about her attempts to discuss firearms with an abusive husband, one Veteran recalled how “whenever we were having an argument, he would go back and check on his guns, take them out and show me he had it, and that pretty much ended the argument, he got his way.”

##### Deferential

Meanwhile, women in the *deferential* category expressed that while they did not *currently* have anything to voice issue about, based on their trusting relationship with their partner, they still felt that they had the ability to voice any future concerns, and that their partner would be receptive and open to such discussions. The ability for women Veterans to express their concerns, have their voices heard, and feel like they had could shape decisions regarding household firearms was a major facet of these relational dynamics and impacted other features described. Nonetheless, as these women Veterans often did not perceive firearm access to be an issue, such conversations were largely hypothetical regarding if either party would engage in any such behaviors (i.e., if the woman Veteran would voice concerns if she had them and, in turn, if her partner would respond to her concerns by changing household firearm use, storage, or access). For example, one Veteran shared that while she was currently indifferent to her husband having a firearm in their home, due to his occupation as a security officer, she was confident that if she had posed a need for him to remove it due to any mental health concerns, “he probably wouldn’t have objected to it. A firearm was not a status symbol for him. It was just a tool for the job he had.”

#### Partner receptivity to the mental health and trauma history of the woman Veteran

Another factor that women Veterans described was their partner’s response (or lack thereof) to them relaying their mental health concerns or trauma histories (e.g., military sexual trauma, IPV). This was another feature that differentiated relational groups.

##### Devalued

Veterans in the *devalued* group felt as if their partner did not care when presented with such issues and that their partner was actively contributing to their distress, sometimes intentionally or maliciously. In explaining such a circumstance, one woman disclosed how, despite repeated attempts to convey the dangers of her having access to firearms, her ex-husband continued to keep his firearm loaded and easily accessible. “There were times where he wouldn’t lock it up. It didn’t go well because he liked to have it on him,” she explained. Continuing, she stated how “there would be times that it would be left out and that would be such a trigger for me, it would be almost panic attack level, like ‘you can’t have this out where I can see it.’ And then he got very angry.” Ultimately, the stress “just got to be too much,” and she took matters into her own hands, “selling it behind his back.”

##### Collaborative and deferential

Conversely, Veterans in both the *collaborative* and *deferential* groups felt as though their partners were (or would be) responsive to their concerns regarding their mental health and trauma histories in relation to household firearm access. For example, a Veteran in a collaborative relationship detailed how she and her partner had worked together to find a solution that worked for them both. “I was battling severe depression and there was a moment I considered using [his firearm]…after that, talking to my ex-husband, we got rid of it.”

#### Impact of presence of children or grandchildren

The impact of children or grandchildren in the home on storage and use of household firearms was another aspect of decision-making between women Veterans and their partners that varied across relational types.

##### Collaborative

For women Veterans in *collaborative* relationships, the presence of children or grandchildren motivated actual, direct changes to household firearms, such as storing them more securely, moving them into gun safes, or even selling firearms that were not considered necessary or of practical use. With children in the home, firearms became a renewed source of discussion. One mother stated that while she and her husband jointly decided to “show them the right way to handle [firearms], let them shoot them, showed them how to clean them,” they both agreed to “never show them any of the combinations or anything about the biometric [safes].” Their logic was simple: “If they’ve been taught, then they’re not curious. And that curiosity is where you get the accidents that happen.”

##### Deferential

Women Veterans in *deferential* relationships noted a stark contrast to their firearm perspectives when the focus shifted from themselves to minors in the household. Specifically, these Veterans, who traditionally might be indifferent to such discussions, indicated that they would be highly motivated to engage their spouse or partner to change household firearm storage, access, or use, if they had concerns about minors accessing household firearms. Similar to women Veterans in *collaborative* relationships, women Veterans in *deferential* relationships indicated that the presence of children (e.g., prospective) would motivate changes in household firearm use or storage. As one Veteran explained, “I’m okay with how we store them. But you know, we’re trying to have a family, and should we bring a child into this world or adopt or whatever the means is, we will 100% get safes.” Veterans in these cases stated that such concerns were heard and their partners agreed to use gun safes to prevent unauthorized access.

##### Devalued

In contrast, while women Veterans in the *devalued* group at times voiced concerns about household firearms due to the presence of children or grandchildren, this did not result in the partner changing household firearm access or storage. Rather, these women Veterans described how their partners were unresponsive to the potential harm that could result from a child accessing an unsecured firearm. Recalling her repeated attempts to discuss with her ex-husband how household firearms were stored, due to her concerns about her daughter’s safety, one woman Veteran explained, “I’d bring it up periodically, but I always made sure that our daughter, she couldn’t get to them. And she knew the guns were there because [my partner] said ‘You don't go here. I've got guns here that could kill you. Don't you ever go there or else you'll get a spanking.’” In another account, a Veteran described how she had always wanted to improve firearm storage in her home—storing ammunition separately from the firearms, if not removing firearms from the home altogether—for her daughter; yet she was not able to do so because of “fear of him.” In some instances, the only thing these Veterans could do to protect themselves and their children was to move themselves and their children out of the home and out of danger. Following an argument in which her husband “pulled out [a handgun] and set it on the dresser,” one Veteran explained how she felt she had to leave with her child, stating how she “wasn’t gonna waste any more time on this. I called my mom and my sister, and they came and brought us back home.”

#### Partner willingness to change firearm storage or use

Partner willingness to make changes to household firearm storage and use was another differentiating factor. Whether due to the Veteran’s stated concerns about how firearms were stored, in response to mental health concerns, or otherwise, women Veterans described their partner’s willingness to make changes to household firearms.

##### Collaborative

Women Veterans with *collaborative* partners often described their partners as open, willing, and capable of making changes to household firearms based on their feedback. One Veteran recounted an event where she had disclosed her mental health concerns to her husband, and her husband’s subsequent actions in removing firearms from the home. “I just expressed to him my bouts of depression, thoughts of wanting to hurt myself, which wasn’t new to him,” she stated. “I just finally told him, ‘I’m telling you this only because of what I need you to do for me. I don’t really wanna talk about it right now, but I will when I’m ready.’ And that was that. And out of respect for it all, he was like ‘Got it. It’s gone.’” In these collaborative relationships, such partners oftentimes had been actively engaged in reducing the Veterans’ access to firearms, whether by removing firearms from the home or changing how they were stored. One Veteran recalled when she had experienced heightened suicide risk; her partner “didn’t ask me any questions or anything else, and we never talked about it. And when I felt as though all of what I was going through wasn’t gonna come back and kill me in my sleep, I was like ‘Okay, when you get the chance, I need to get that box back from you.’ And that was the extent of the conversation.”

##### Deferential

Similarly, women Veterans who were largely *deferential* about the current state of household firearms expressed that if they did have concerns, their partners would be willing to make those changes. For these Veterans, these statements were largely hypothetical, rooted in the longstanding trust they had developed with their partners. When asked if her partner would be willing to make changes if she were to be at risk for suicide, one woman explained how she believed he would because “I trust him. But I think it’s the individual and who they trust the most…because not everyone has a husband they trust as much as I trust mine, you know?”.

##### Devalued

Women Veterans in *devalued* relationships, however, expressed that their partners felt little to no obligation to make such changes. Despite repeated pleas to their partners, few women Veterans expressed any real sense that their partner would take steps in changing how firearms would be stored in the home. For example, one Veteran detailed how despite an ex-husband knowing of her history of suicide attempts, he made little effort to change how firearms were stored around their home. “[The pistol] wasn’t locked in there or anything. He was kind of worried that I was gonna kill myself…and when I think back on it, it’s kind of messed up that he was worried about me killing myself but didn’t lock it anywhere.”

#### Women Veterans’ desire to involve partners in firearm LMSC

Women Veterans in each relational type varied in their desire and preference regarding including their partners in firearm LMSC. Their desire for whether to include their partners in firearm LMSC were largely influenced by their level of trust in their partner, their perceptions of their partner, and their prior experiences discussing firearms with their partner.

##### Collaborative

Women Veterans in *collaborative* relationships expressed a desire to include their partners as collaborators in firearm LMSC. In many instances, their prior positive experiences discussing firearms with their partners, making joint decisions together, and their partner’s supportive responsive to their concerns facilitated this preference. “We talk about firearms a lot and I’m confident and I trust him. I know what his ultimate goal is. It’s for the protection of the family and I trust him,” one Veteran shared, noting that this shared trust and engagement in firearm-related issues would make her partner a valued collaborator in LMSC. Because of this shared trust, she indicated that if she were to be at elevated risk for suicide, she would “talk to (him) about it first…my husband and I are very proactive medically.”

##### Deferential

Similarly, women Veterans in *deferential* relationships indicated that they would be open to including their partners in firearm LMSC. For example, when asked if her partner would be willing to make changes if she were to be at risk for suicide, one woman explained how she believed he would because “I trust him. But I think it’s the individual and who they trust the most…because not everyone has a husband they trust as much as I trust mine, you know?”.

##### Devalued

Women Veterans in *devalued* relationships, however, expressed no desire to include their partners in firearm LMSC. Rather, they indicated that including their partners in firearm LMSC was undesired, inherently problematic, and likely unsafe due to IPV and the partner’s domineering behavior to themselves, as well as to their children. Thus, women Veterans in *devalued* relationships clearly indicated that controlling partners and those who use IPV would be unacceptable collaborators in firearm LMSC. Describing a past, devalued relationship, a woman Veterans indicated that she did not view her ex-husband as trustworthy enough to involve in firearm LMSC, due to his unsafe firearm practices and behaviors: “[Having firearms in the home] didn’t feel good, and I made it clear to him that we would have to get rid of all of them if we ever started having children because I didn’t think he’d ever keep it safe,” later detailing how “he would be the one that would leave the safe door unlocked for a minute.”

## Discussion

Reducing access to potentially lethal means of suicide is one of the core tenets of suicide prevention. Encouraging such practices is important when working with women Veterans at elevated risk for suicide, as firearm injury has become the leading method of suicide among members of this population (U.S. Department of Veterans Affairs and Office of Mental Health and Suicide Prevention 2022a). For a significant portion of women Veterans, their firearm access is through other household members, rather than solely through firearms that they personally own (Cleveland et al. 2017; Monteith et al. 2022b). This, combined with high rates of IPV among women Veterans (Iverson et al. 2017), where firearms may be used to threaten or intimidate them, complicates efforts to limit firearm access during periods of elevated suicide risk. Yet firearm LMSC has been developed with the assumption that counseled patients are able to influence household firearm decisions regarding access and storage. However, our findings indicate that, in some cases, women Veterans’ decisional authority regarding household firearms and their storage is limited.

Descriptions of collaborative, devalued, and deferential relational types highlight the many nuances of how this decision authority is manifested and variable. Building upon prior studies (Monteith et al. 2020), our findings underscore the important role of women Veterans’ partners in women Veterans’ household firearm access. Recognizing these relational types and the facilitators and barriers of each may help healthcare providers to tailor LMSC recommendations to individual woman Veterans’ situation and needs. Further, the nature of these relationships appear to influence women Veterans’ willingness and ability to change how household firearms are stored, as well as their desire to include their partners in firearm LMSC. It is therefore essential that healthcare providers understand both the *nature* of such relationships, as well as the desire of the patient to include their partner in LMSC conversations and implementation of associated recommendations.

For women Veterans in *collaborative* relationships, there are numerous facilitators to including their partners in firearm LMSC, such as shared decision-making and trust. As a result, including these partners in firearm LMSC conversations is likely feasible and may be beneficial when the partner owns household firearms or when the woman Veteran desires additional support in reducing her access to personally owned firearms. At the same time, many women Veterans in collaborative relationships indicated that they had already discussed household firearm access and storage with their partners, in a manner that (per the Veteran’s account) was acceptable to both. For example, in some instances, partners had helped to reduce the Veteran’s access to firearms during times of heightened suicide risk. Thus, barriers to including collaborative partners in firearm LMSC appear to be low. Consequently, it is unsurprising that women Veterans in *collaborative* relationships expressed a desire and willingness to include their partners in LMSC. Nonetheless, as we only analyzed data from women Veterans (and not from their partners) in this study, it will be important to also obtain the perspectives of partners who are in collaborative relationships to ensure that they also have the resources needed to support their partners in firearm LMSC.

In comparison, for healthcare providers working with women Veterans in *deferential* relationships, including partners in LMSC is likely more important if there are concerns regarding the woman Veteran’s firearm access (for example, if she is experiencing increased risk for suicide). This is because women Veterans in such relationships were rarely the owners of household firearms, did not have strong preference on how firearms were used or stored, and did not collaborate with their partners on decision-making regarding household firearms. Rather, they trusted their partners to make household firearm decisions, and thus, tended to defer decisions regarding household firearms to their partners, typically forgoing explicitly discussing and addressing firearm safety with their partners. Yet, these Veterans were open to having such discussions if needed in the future—for example, if mental health concerns or the presence of children in the home would prompt dissatisfaction or concerns regarding household firearms. Several aspects of deferential relationships also support the potential feasibility of including these Veterans’ partners in firearm LMSC, such as these women Veterans’ trust of their partners and the compassion and openness of the partner when the Veteran disclosed prior trauma or mental health concerns. While women Veterans in deferential relationships were confident that their partners would be supportive in changing household firearm storage and access if needed, this was largely hypothetical. Should such conversations progress differently than expected, clinician support in navigating such conversations may be needed.

*Devalued* relationships likely pose the most challenges to firearm LMSC. This is due to the lack of shared decision-making between the women Veteran and her partner, power imbalances, IPV, and in such instances, fear among women Veterans to suggest or continue advocating for changes to household firearm access and storage due to prior violence or threats of violence to them or their children. In these circumstances, partners controlled women Veterans’ access to household firearms and dictated decisions about access, use and storage for the entire household, even when doing so was dangerous for both women Veterans and children in the household. When women Veterans voiced their concerns, whether due to their own trauma histories, their mental health, or fear of their children gaining access to firearms, their concerns were ignored, neglected, or minimized. Moreover, some partners used firearms as a means of intimidating their women Veteran partners to get what they wanted (e.g., to shut down an argument). Thus, including partners using these invalidating dynamics in firearm LMSC is likely contraindicated and is inconsistent with women Veterans’ stated preferences. Obtaining couples counseling to address broader relational dynamics may be indicated, although the effectiveness and safety of pursuing this option varies considerably (Karakurt et al. 2016). Relationships marked by high levels of coercion and control or physical violence are not appropriate for conjoint approaches given concerns for women’s physical and emotional safety. For these women Veterans, ensuring their personal safety and that of any children living in the household is vital; for the woman Veteran in these dynamics, risk of injury and homicide is a critical concern (Campbell et al. 2007). The role of healthcare providers in such circumstances may entail validation of the woman Veteran’s experiences, psychoeducation, and safety planning for if violence escalates (Doyle et al. 2022). Additionally, providers should consider providing or referring women experiencing IPV to more comprehensive advocacy services and counseling interventions focused on enhancing women Veterans’ safety, empowerment, and mental health. A novel intervention called “Recovering from IPV through Strengths and Empowerment” demonstrated meaningful gains in self-efficacy, empowerment, and depression, all of which are prospectively associated with increased safety and improved mental health (Webermann et al. 2022; Dardis et al. 2018; Iverson et al. 2011).

While we suggested clinical considerations for working with women Veterans experiencing each of these relational types, future research is warranted to determine if these are indeed correct and to elucidate evidence-based strategies for preventing suicide by firearm among women Veterans in each of these groups. This is particularly important for women Veterans in relationships where their partner devalues their role in firearm decision-making and where IPV is also present. Another important area for future research, which our team is pursuing, is understanding women Veterans’ partners’ perspectives, experiences, and needs regarding firearm LMSC. This is necessary to obtain a comprehensive understanding of experiences, perspectives, barriers, and facilitators to firearm LMSC between women Veterans and their partners. Lastly, quantitative research to validate and/or refine these three relational types and examine associations with other constructs would be valuable.

### Limitations

Study limitations include the focus on women Veterans who used VHA services and had a lifetime history of suicidal ideation and/or suicide attempt, as well as the median age of 53 and low proportion of Veterans with current parenting responsibilities or minors in the home. This may limit the relevance of findings to women Veterans outside of VHA care, without a history of suicidality, and those younger with parenting responsibilities. In addition, while the sample was racially and ethnically diverse, low numbers of Asian American Veterans and the absence of any Pacific Islander Veterans is a limitation, particularly given increasing suicide rates within that population (U.S. Department of Veterans Affairs and Office of Mental Health and Suicide Prevention 2022a). Further, while purposeful sampling was undertaken and the sample size relatively large for a qualitative study, the small sample size, inductive approach, and qualitative interview design precludes generalizability to the full woman Veteran population. Lastly, we were not able to quantitively examine if and how participant characteristics (e.g., IPV, PTSD, parenting or marital status) differed by relational types, which will be an important undertaking for future research.

## Conclusion

A critical aspect of preventing suicide by firearm among women Veterans with household firearm access entails understanding the vast range of agency among women Veterans over household firearm decisions, even when they are at elevated risk of suicide. Considering the relational dynamics between women Veterans and their partners may further promote delivery of tailored, appropriate LMSC strategies, especially within the context of IPV.

## Data Availability

Data utilized in this study cannot be publicly shared because of privacy and confidentiality requirements. Data are available from the VA R&D Committee (phone contact: 303-399-8020) for researchers who meet the criteria for access to confidential study data.
